# Improving the Precision of Our Ecosystem Calipers: A Modified Morphometric Technique for Estimating Marine Mammal Mass and Body Composition

**DOI:** 10.1371/journal.pone.0091233

**Published:** 2014-03-10

**Authors:** Michelle R. Shero, Linnea E. Pearson, Daniel P. Costa, Jennifer M. Burns

**Affiliations:** 1 Department of Biological Sciences, University of Alaska Anchorage, Anchorage, Alaska, United States of America; 2 School of Fisheries and Ocean Sciences, University of Alaska Fairbanks, Fairbanks, Alaska, United States of America; 3 Department of Ecology and Evolutionary Biology, University of California Santa Cruz, Santa Cruz, California, United States of America; University of St Andrews, United Kingdom

## Abstract

Mass and body composition are indices of overall animal health and energetic balance and are often used as indicators of resource availability in the environment. This study used morphometric models and isotopic dilution techniques, two commonly used methods in the marine mammal field, to assess body composition of Weddell seals (*Leptonychotes weddellii*, *N* = 111). Findings indicated that traditional morphometric models that use a series of circular, truncated cones to calculate marine mammal blubber volume and mass overestimated the animal’s measured body mass by 26.9±1.5% SE. However, we developed a new morphometric model that uses elliptical truncated cones, and estimates mass with only −2.8±1.7% error (*N* = 10). Because this elliptical truncated cone model can estimate body mass without the need for additional correction factors, it has the potential to be a broadly applicable method in marine mammal species. While using elliptical truncated cones yielded significantly smaller blubber mass estimates than circular cones (10.2±0.8% difference; or 3.5±0.3% total body mass), both truncated cone models significantly underestimated total body lipid content as compared to isotopic dilution results, suggesting that animals have substantial internal lipid stores (*N* = 76). Multiple linear regressions were used to determine the minimum number of morphometric measurements needed to reliably estimate animal mass and body composition so that future animal handling times could be reduced. Reduced models estimated body mass and lipid mass with reasonable accuracy using fewer than five morphometric measurements (root-mean-square-error: 4.91% for body mass, 10.90% for lipid mass, and 10.43% for % lipid). This indicates that when test datasets are available to create calibration coefficients, regression models also offer a way to improve body mass and condition estimates in situations where animal handling times must be short and efficient.

## Introduction

Establishing links among variations in environmental conditions, prey availability, foraging success, and population status has become increasingly important as ecosystems face climate and anthropogenic threats. While monitoring ecosystem processes can be difficult, changes in the mass and body condition of apex predators can be used as indices of ecosystem health [1]–[5]. Accurate estimates of body mass and condition are also essential for a wide range of ecological and physiological studies, as they represent animals’ net energetic costs or gains [6]–[8]. In addition to being a proxy for overall animal health and fitness, in marine mammals, body composition also influences animal streamlining, buoyancy, metabolic demand, and thermoregulatory costs [9]–[14].

In fieldwork situations, mass and body composition (e.g., lipid stores) can be most directly measured by weighing animals and using hydrogen-isotope dilution techniques, respectively. Isotopic dilution methods measure the animal’s total body water (TBW) volume by allowing a bolus of labeled water to dilute within the body water pool. Measured TBW volume, coupled with estimates of the hydration state of lean tissue (73% water) and adipose (10% water), allows for relatively accurate (mean error: 3.7%) estimates of body composition in mammals [7], [15], [16]. Errors arise from the generation of metabolic water, exchange of isotope with non-aqueous hydrogen ions, dilution in stomach water, and evaporative water loss [6], [16], [17]. Method accuracy is also influenced by errors in the assumed hydration state of body tissue, as water content in the blubber and lean tissue may differ by species, season, and age [18]–[20]. Additionally, validations of isotopic dilution to true TBW and lipid stores by desiccation and dissection comparisons have only been performed in a select number of studies because these destructive methods are so labor intensive [21]–[25]. Still, isotopic dilution methods have been used in a wide range of species, including pinnipeds, and are generally assumed to be the “golden standard.” Despite the potential sources of error, isotopic dilution has the advantage of accounting for both the subcutaneous and internal lipid stores, and in most study systems, body composition determined by these methods is considered to be the most reliable field measure of total body lipid content [6], [20].

However, isotopic dilution protocols can be logistically difficult, costly, and time consuming. As a result, a wide variety of proxy variables have been identified to serve as indicators of marine mammal body condition. Because marine mammals store large amounts of energy in their large subcutaneous blubber layer, these simpler methods have placed a large emphasis on blubber volume. Proxies range from models using a single length and girth measurement to estimate body mass, to a single blubber depth or bioelectrical impedance analysis to indicate animal body condition [25]–[28]. As these overly simplistic models are often poor predictors of body composition, Gales & Burton [29] outlined a technique for determining blubber volume in pinnipeds. Using morphometric (lengths and girths) and ultrasound blubber depth measurements, the animal’s body shape is reconstructed as a series of circular truncated cones. Blubber and core tissue density estimates are then used to convert the calculated volumes of the cones to blubber mass. This truncated cone method has been used to determine blubber mass in multiple pinniped [8], [9], [11], [30], [31] and cetacean species [32], [33]. However, soon after this method was developed, Slip et al. [34] described the phenomenon of “fat slumping” wherein gravity causes blubber along the lateral sides of the animal to “slump” and, therefore, causes the animal’s true shape to deviate from circular towards elliptical.

Since Gales & Burton [29], many studies have used modeled estimates of blubber volume to calculate body composition, despite the fact that blubber mass measurements are not equivalent to lipid mass. Adult marine mammal blubber contains structural proteins and is not composed entirely of lipid. Blubber lipid content ranges from ∼30−95%, depending on species, reproductive status, overall health, and season [19], [35]–[38]. Still, very few studies incorporate actual blubber lipid content into calculations [31], [39]. In addition, pinniped studies that solely use the truncated cones method as a measure of condition cannot account for internal lipid stores. Thus, potential sources of error should be acknowledged when evaluating the success with which a morphometric technique can determine body mass and condition.

However, morphometric models are so attractive because studies at the population level require large sample sizes to detect potentially small but significant changes in mass and condition. For large animals such as marine mammals, determining body mass via direct weighing is difficult, and isotopic dilution requires long sedation and equilibration times. Morphometric models thus offer a good alternative, yet very few studies have attempted to construct predictive models that employ only a few non-invasive measurements to estimate body mass and condition [34], [40].

This study compared methods of estimating Weddell seal (*Leptonychotes weddellii*) body composition, including morphometric models and isotopic dilution techniques. In addition, we developed a modified truncated cone method that accounts for blubber and core body slumping by modeling animal cross-sectional shape as ellipses instead of circles, and compared accuracy of body mass and condition estimates. Then, we developed models to estimate body mass and composition from a few non-invasive morphometric measurements. Our findings provide a quantitative basis for choosing efficient and logistically feasible methods of assessing marine mammal body mass and condition under constrained field conditions.

## Materials and Methods

### Ethics Statement

Animal handling protocols were approved by the University of Alaska Anchorage and University of California Santa Cruz’s Institutional Animal Care and Use Committees. Research and sample import to the United States was authorized under the Marine Mammal permit No. 87-1851-04 issued by the Office of Protected Resources, National Marine Fisheries Service. Research activities were approved through Antarctic Conservation Act permits while at McMurdo Station.

### Animal Capture

Adult Weddell seals (*N* = 111; Table 1) were captured on fast-ice in Erebus Bay (∼77°S, 165°E) and the Victorialand coastline (∼76°S, 162°E), Antarctica from 2010−2012. Animals were handled in Jan/Feb (Austral fall) after the molt period when seals are typically in their poorest condition (lowest lipid stores) and in Oct/Nov (Austral spring; pre-breeding period) after the animals have been actively foraging for ∼8 months [41].

**Table 1:** Sample sizes and means ± SE for body mass and composition.

Season	Reproductive Status	Total Body Mass (M_T_; kg)	TBW (%M_T_)	Lipid by HTO (%M_T_)	Blubber Biopsy Lipid Content (%)
*Jan/Feb*	Skip-Breeding Female	320.7±10.3 (*52*)	50.7±0.5 (*32*)	30.5±0.7 (*32*)	79.3±1.3 (*49*)
	Male	231.8±12.5 (*10*)	50.2±0.7 (*7*)	31.4±1.1 (*7*)	86.2±2.2 (*5*)
*Oct/Nov*	Non-Reproductive Female	335.8±14.2 (*28*)	45.9±0.6 (*22*)	37.6±0.8 (*22*)	83.5±1.4 (*24*)
	Reproductive Female	413.7±13.3 (*16*)	46.4±0.6 (*13*)	36.8±0.9 (*13*)	84.1±1.9 (*17*)
	Male	294.6±11.7 (*5*)	47.5±1.2 (*2*)	35.4±1.9 (*2*)	---
*Overall*	All	328.8±7.6 (*111*)	48.5±0.4 (*76*)	33.9±0.6 (*76*)	81.6±0.9 (*95*)

Mean ± SE total body mass (M_T_), total body water (TBW) and lipid stores as determined by isotopic dilution (as %M_T_), and lipid content of blubber biopsies (% wet mass) for animals handled throughout this study. Animals are classed by season and reproductive status, and sample sizes are in parentheses.

Animals were sedated with an initial intramuscular dose of 1.0 mg·kg^−1^ tiletamine/zolazepam HCl, followed by intravenous injections of ketamine and diazepam (1∶1 ratio; 100 mg·mL^−1^ and 5 mg·mL^−1^) as necessary.

### Direct Measures of Total Body Mass and Lipid Stores

Total body mass (M_T_) was determined by direct weighing (MSI-7200-1T Dyna-Link digital dynamometer, capacity 1,000±1.0 kg).

**Figure 1:**
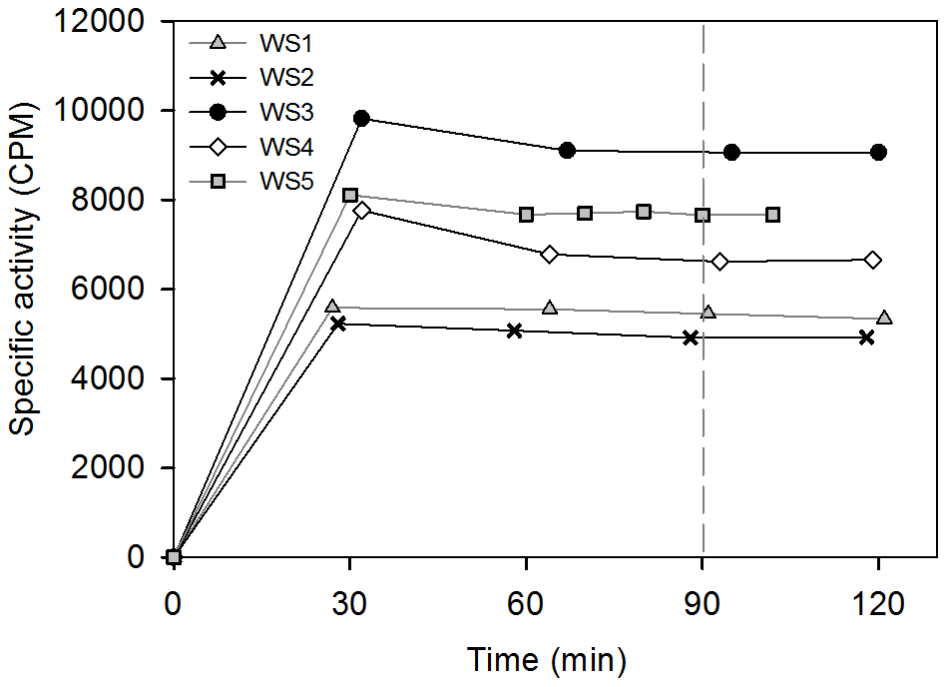
HTO equilibration curve for five Weddell seals showing plateau by 90 min.

The body composition of 76 animals (Table 1) was determined by isotopic dilution. Following collection of an initial blood sample, 1−1.5 mCi tritiated water (HTO) was injected into the extradural vein. Each syringe was gravimetrically calibrated prior to use, and syringes were flushed with blood after injection to ensure complete administration of the dose. Blood was collected from the extradural vein in serum separator vacutainers at 15−30 min intervals for 90−120 min post-injection. Serum was separated from whole blood samples via centrifugation and stored at −80°C until analysis. Water was extracted from samples in triplicate using the freeze-capture technique as described in Ortiz et al. [15]. HTO specific activity (counts per minute; CPM) was determined using a Packard Tri-Carb 2900TR liquid scintillation counter (Packard Bioscience Co., Meriden, CT) by adding 100 µL distillate into 10 mL ScintiSafe scintillation cocktail (Fisher Scientific, Inc.). Each of the triplicate distillate samples was counted twice for 20 min (∼10,000−20,000 total counts). Triplicates were only accepted if CV’s were <2%. Pre-injection activity determined for each animal was subtracted from all post-injection activities. Injectate standards were distilled in six replicates before and after analyzing animal serum samples to ensure minimal intra-assay variation. Dilution curves plateaued by 90 min (Fig. 1), and total body water (TBW) was calculated as:
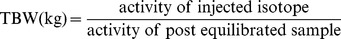
(1)


TBW values were reduced by 3.3% to account for post-injection isotope losses due to exchanging hydrogen ions and ventilation [42]. Total body lipid mass (TBL_HTO_) was calculated from TBW following Reilly & Fedak [24]:
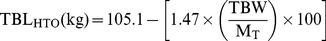
(2)


where M_T_ is total body mass, and both TBW and M_T_ are in kg. Isotopic dilution techniques yielded total lipid mass, regardless of its location subcutaneously or internally, and lean mass was considered to be fat free tissue.

### Truncated Cone Estimates of Body Mass and Composition

**Figure 2:**
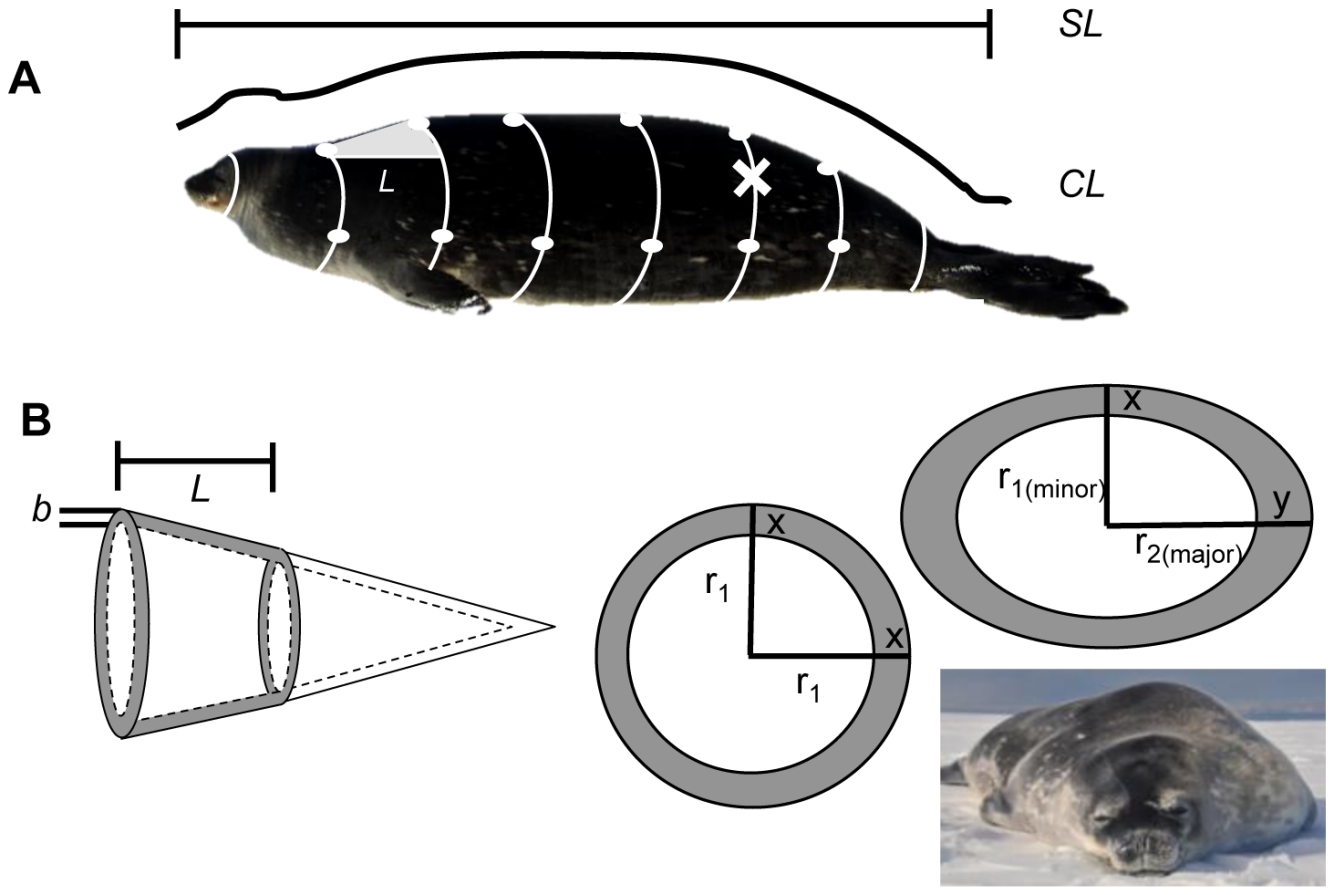
Morphometric measurements taken for each study animal. (**A**) *SL* = Standard length, *CL* = Curve length, Girths =  white lines, Blubber depths =  white dots, Cone section length calculations =  grey triangle and “L”. Site of blubber biopsy is marked with “X”. (**B**) Reconstruction of truncated cones with segment length “*L*” and blubber depth “*b*” (*At left*). Circular and elliptical cross-sections shown (*At right*). Because an ellipse has a major and minor radius “r,” the model can account for different dorsal and lateral blubber depths (x and y) and more accurately reflect true animal shape.

After animals were captured and weighed, a series of morphometric measurements were taken in order to model mass and body composition: girths, straight lengths (sLengths), and curvilinear lengths (cLengths) measured from the animal’s nose to eight consecutive sites along the body (Fig. 2A). Subcutaneous blubber thickness was measured at six dorsal and lateral sites (Fig. 2A) using a SonoSite Vet180Plus portable ultrasound and 3.5 MHz convex transducer (SonoSite Inc., Bothell, Washington, USA) while the animal was in sternal recumbency. Blubber depth measurements were used to calculate blubber volume and mass using the traditional truncated cones method as described by Gales & Burton [29]. The animal’s body shape was reconstructed as a series of circular truncated cones, and blubber volume was calculated as the volume of the outer cone (total body volume) minus the volume of the inner cone (core body volume; Fig. 2B). The volume of the head and tail were estimated using full cones composed entirely of lean mass, while flippers were not included in truncated cone models (Table 2) because there is very little blubber around the head, tail, fore-, and hind-flippers [43].

**Table 2:** Calculation of subcutaneous fat for seal WS12-22 using traditional truncated cones with circular cross-sections.

		Outer Cone Measurements					Inner Cone Measurements		Body Volume			Mass Conversions		
Cone Position		Girth (cm)	Radius (Outer; cm)	Radius Difference (cm)	Curvilinear Length (cm)	Straight Length (cm)	Average Blubber Depth (cm)	Radius (Inner; cm)	Outer Cone (L)	Inner Cone (L)	Blubber (L)	Core (kg)	Blubber (kg)	Total Body (kg)
**1**	**nose**	0	0	0	0	0	0	0	0	0	0	0	0	0
**2**	**nose → ears**	75	11.9	11.9	20	16.1	0	11.9	2.4	2.4	0	2.6	0	2.6
**3**	**ears → neck**	137	21.8	9.9	27	25.1	5.99	15.8	23.1	15.3	7.8	16.8	7.3	24.2
**4**	**neck → axillary**	202	32.2	10.4	37	35.5	6.13	26.0	82.2	49.8	32.4	54.8	30.5	85.3
**5**	**axillary → sternum**	121	33.7	1.6	31	31.0	5.64	28.1	105.6	71.3	34.3	78.4	32.3	110.7
**6**	**sternum → middle**	206	32.8	1.0	38	38.0	5.32	27.5	132.1	92.1	39.9	101.3	37.5	138.9
**7**	**middle → umbilicus**	181	28.8	4.0	37	36.8	5.48	23.3	109.8	74.7	35.1	82.2	33.0	115.1
**8**	**umbilicus → pelvis**	127	20.2	8.6	42	41.1	4.55	15.7	78.4	49.7	28.7	54.7	26.9	81.6
**9**	**pelvis → ankles**	76	12.1	8.1	23	21.5	0	12.1	18.0	13.1	4.9	14.4	4.6	19.0
**10**	**ankles → tail**	0	0	12.1	21	17.2	0	0	2.6	2.6	0	2.9	0	2.9
								**Total:**	**554.2**	**371.0**	**183.1**	**408.2**	**172.1**	**580.3**
											**Measured Mass (kg):**			**451.0**
											**Error from Measured Mass:**			**28.7%**

Radius outer cone = Girth/2π;

Radius difference = base − roof of cone;

Straight Length = sqrt(Curvilinear Length^2^ − Radius Difference^2^);

Radius inner cone = Radius outer cone – (2 × Blubber Depth);

Outer/Inner Cone Volume = (1/3)π × [(Radius Outer/Inner_1_)^2^ + (Radius Outer/Inner_1_ × Radius Outer/Inner_2_) + (Radius Outer/Inner_2_)^2^];

Blubber Volume =  Outer Cone Volume − Inner Cone Volume;

Mass Conversions: Core Mass =  Core Volume × 1.1; Blubber Mass =  Blubber Volume × 0.94; Total Body Mass =  Core Mass + Blubber Mass.

Because seals did not appear circular in cross-section when lying on the ice, the procedure was adjusted to include measurements of animal height and width at each site along the body for both outer and inner body cones (*N* = 11). The circular truncated cones calculations [29] were then modified for elliptical body cross-sections in animals for which all measurements were taken (*N* = 10; Fig. 2B; Table 3). In the modified elliptical cone method, the straight length for each truncated cone segment was calculated by using right triangles along the animal (Fig. 2A), with the measured curvilinear length as the hypotenuse and half the height difference between cone segments as the adjacent side of each triangle:
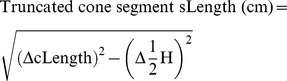
(3)


**Table 3:** Calculation of subcutaneous fat for seal WS12-22 using truncated cones with modified, elliptical cross-sections.

		Outer Cone Measurements						Inner Cone Measurements				Body Volume			Mass Conversions		
Cone Position		Body Height (cm)	Body Width (cm)	Minor Radius (Outer Cone; cm)	Minor Radius Difference (cm)	Curvilinear Length (cm)	Straight Length (cm)	Dorsal Blubber Depth (cm)	Lateral Blubber Depth (cm)	Diameter Minor Axis (cm)	Diameter Major Axis (cm)	Outer Cone (L)	Inner Cone (L)	Blubber (L)	Core (kg)	Blubber (kg)	Total Body (kg)
1	nose	0	0	0	0	0	0	0	0	0	0	0	0	0	0	0	0
2	nose → ears	20	33	10.0	10.0	20	17.3	0	0	20.0	33.0	3.0	3.0	0	3.3	0	3.3
3	ears → neck	27	57	13.5	3.5	27	26.8	5.25	6.72	16.5	43.6	22.5	14.5	8.0	15.9	7.5	23.4
4	neck → axillary	42	73	21.0	7.5	37	36.2	5.10	7.15	31.8	58.7	64.3	35.5	28.8	39.1	27.0	66.1
5	axillary → sternum	44	81	22.0	1.0	31	31.0	5.57	5.71	32.9	69.6	80.6	50.4	30.1	55.5	28.3	83.8
6	sternum → middle	41	85	20.5	1.5	38	38.0	5.82	4.82	29.4	75.4	105.1	67.1	38.0	73.8	35.7	109.5
7	middle → umbilicus	34	68	17.0	3.5	37	36.8	5.68	5.28	22.6	57.4	83.3	50.2	33.0	55.3	31.1	86.3
8	umbilicus → pelvis	30	45	15.0	2.0	42	42.0	4.67	4.42	20.7	36.2	59.6	33.3	26.3	36.6	24.7	61.4
9	pelvis → ankles	12	35	6.0	9.0	23	21.2	0	0	12.0	35.0	14.0	9.6	4.4	10.5	4.1	14.7
10	ankles → tail	0	0	0	6.0	21	20.1	0	0	0	0	2.2	2.2	0	2.4	0	2.4
											**Total:**	**434.6**	**265.8**	**168.6**	**292.4**	**158.6**	**451.0**
														**Measured Mass (kg):**			**451.0**
													**Error from Measured Mass:**				**0.0%**

Minor radius outer cone = Body Height/2;

Minor radius difference = base − roof of cone minor radius;

Straight Length = sqrt(Curvilinear Length^2^ – Minor Radius Difference^2^);

Diameter inner cone major/minor axis =  Body Height/Width – (2 × Blubber Depth);

Outer/Inner Cone Volume =  [(Straight length × π)/12] × [D_1_D_2_ + D_3_D_4_ + sqrt(D_1_D_2_D_3_D_4_)];

Blubber Volume =  Outer Cone Volume − Inner Cone Volume;

Mass Conversions: Core Mass =  Core Volume × 1.1; Blubber Mass =  Blubber Volume × 0.94; Total Body Mass =  Core Mass + Blubber Mass.

where ΔcLength is the curvilinear length of the elliptical truncated cone segment and Δ½H is the height difference from the center of the frustum (half the animal height) for that segment of the animal. The volume of the total body outer elliptical truncated cone was calculated as: 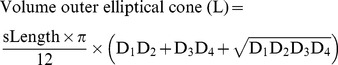
(4)


where D_1_ and D_2_ are the major (measured animal width) and minor (height) diameters of the anterior end of the cone segment, and D_3_ and D_4_ are the major and minor diameters of the posterior end of the cone segment, respectively. The summation of these elliptical truncated cone segments yielded total body volume. To determine the volume of the animal’s inner core, blubber depths were subtracted from the body diameter:
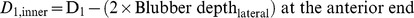
(5)

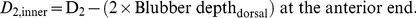
(6)


The same equations were used to find D_3, inner_ and D_4, inner_ at the posterior end of the cone segment. Once blubber depths were subtracted, Eqn (4) was used to calculate the volume of the inner core for each truncated cone segment using these modified diameters. Summation of the core truncated cones yielded core body volume, and blubber volume was calculated as the difference between the outer and inner core volumes:
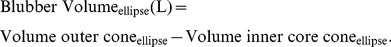
(7)


In both circular and elliptical truncated cone models, blubber and core volume estimates were converted to body mass by assuming that the lean body core and blubber layer had densities of 1.1 g·mL^−1^ and 0.94 g·mL^−1^, respectively [29], [30], [44]. Blubber and total body mass were estimated by summing the mass of each truncated cone segment, and the head and tail cones.

Blubber mass (BM) calculated using elliptical truncated cones (BM_E_) was regressed against blubber mass estimated from traditional circular cones (BM_C_). This relationship was highly significant, and the regression equation was used to estimate BM_E_ for animals where it could not be directly calculated.

### Blubber Lipid Content

A blubber biopsy was taken from each animal after the site was prepped with Betadine and Lidocaine, using a sterile 6-mm biopsy punch just below the midline at the umbilicus (Fig. 2A), flash frozen and stored at −80°C. To compare lipid content from the single biopsy site to average values across the body, blubber was collected opportunistically from a female that died of natural causes in McMurdo Sound, <24 hrs post-mortem, from all 12 sites where blubber depth was measured for truncated cone models.

Full thickness blubber biopsies were weighed to the nearest 0.001 g and lipid content of the samples was determined gravimetrically after extracting lipids using a 2∶1 chloroform-methanol rinse in a Soxhlet apparatus [45], [46]. In the event that blubber lipid content was not available for a particular animal, the average lipid content for that season and reproductive class was used to convert BM to lipid mass (Table 1). Lipid mass within the blubber layer (BLM) was determined for elliptical (BLM_E_) and circular truncated cones (BLM_C_) using BM determined by morphometric models, as described above, and the lipid content per unit mass in the blubber biopsy:
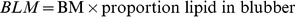
(8)


### Statistical Analyses

Prior to statistical analyses, data were assessed for outliers and normality. Body composition data were normally distributed and between 20−80%, and thus were not arcsine transformed. Results are reported as mean ± SE. To determine whether animals were indeed elliptical in cross-section, width:height ratios were compared to that of a circle (width:height  = 1) using one-sample t-tests, while a two-way ANOVA was used to assess differences in the width:height ratio across the body, and between the inner and outer cones (*N* = 11). Paired t-tests were used to determine whether total body mass estimates derived from the circular truncated cone method differed from actual body mass (M_T_). Repeated-measures ANOVAs with Bonferroni post-hoc tests were used to determine whether mass and body composition estimates from both elliptical (*N* = 10) and circular (*N* = 76) truncated cone methods differed from the direct measurement of M_T_ or TBL_HTO_. Regression analyses were used to determine significant relationships between calculated BM or BLM, and TBL_HTO_ (*N* = 76).

To create models that maximized the *R* ^2^ and minimized root-mean-square-error (RMSE; equivalent to standard deviation), forward stepwise multiple regression models were used to estimate body mass and condition. M_T_ was estimated from straight length (sLength) and the square of axillary girth (LG^2^) measurements to compare models to the simplest published methods [26], [27], and also estimated using the suite of lengths and girths measured during this study (*N* = 111). Animals for which all lengths, girths, and blubber depths could be measured in addition to TBL_HTO_ (*N* = 76) were used to create regression models relating morphometric measures to body composition. TBL_HTO_ was estimated with and without true M_T_ included in models.

Second-order Akaike information criterion (AICc) tests were implemented using the R “MuMIn” package to select the best models, incorporating the fewest number of parameters, as would be useful if animal handling times in the field are constrained. Variables were only added to models when the ΔAICc of the added parameter was ≥ 2. Season, sex, and reproductive status were added as categorical “dummy” variables. If a categorical variable was an important parameter in the model, it was added to all regressions as this would be a known parameter in a fieldwork situation. When incorporating parameters into predictive models, multicollinearity was assessed by variance inflation factors (VIF); all were less than 7. While lower than a VIF of 10, which is typically considered to be a concern [47], [48], to further ensure that added parameters were not a spurious result of multicollinearity, RMSE of models was determined using k-fold cross-validations (with 10 folds) using the R “DAAG” package. Parameters were only added to the model when RMSE decreased. All analyses were conducted in R (v 2.15.2) and significance was assessed at the 95% level (*P*<0.05).

## Results

### Morphometric Estimates of Body Mass versus Weighing

Animals in this study varied widely in M_T_ (181−502 kg), TBW (97.6−253.0 kg; 40.5−56.5% M_T_), and TBL_HTO_ (54.3−186.0 kg; 22.0−45.5% M_T_) measurements (Table 1).

**Figure 3:**
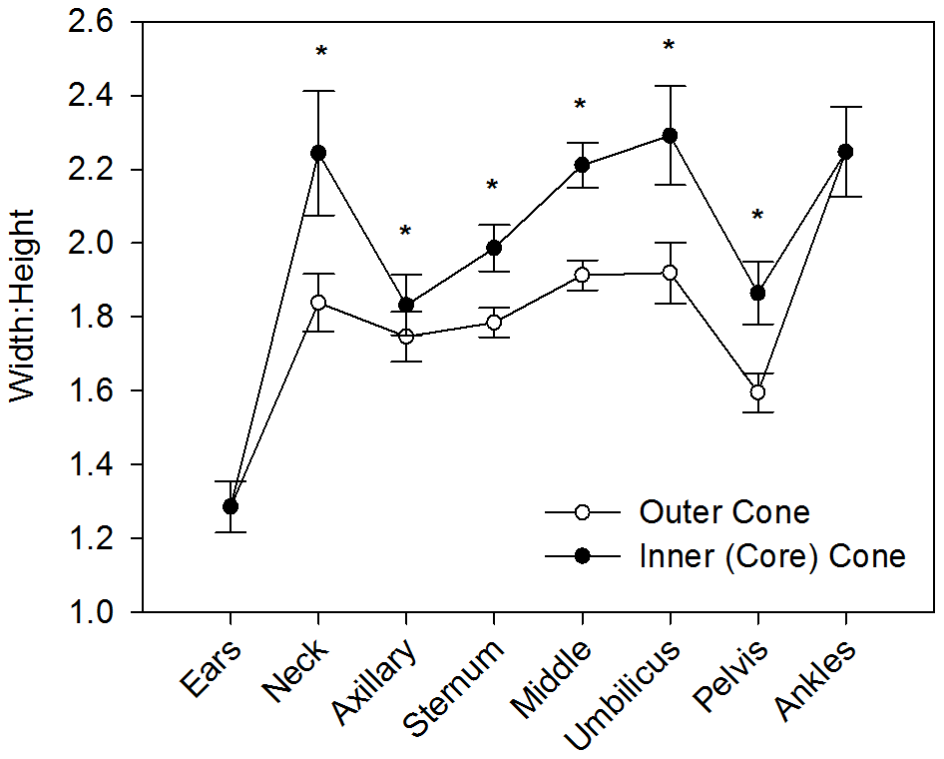
Weddell seal body cross-sections are elliptical. Mean ± SE width-to-height ratios along the body of adult female Weddell seals (*N* = 11), with a circle having a ratio = 1. *Asterisk* indicates that the width-to-height ratio of the inner core cone is significantly greater than the outer, total body cone.

Animals were elliptical-shaped in cross-section as indicated by width:height ratios >1 at all eight sites along the body (Fig. 3; One-sample t-tests- ears: *t*_10_ = 4.114, *P* = 0.002; neck to ankles: all *t*_10_ > 10, all *P*<0.001). Width:height ratios differed by site along the body (Fig. 3; Two-Way ANOVA- *F*_7, 160_ =  20.010, *P*<0.001) and, once blubber depths were subtracted, the inner core cone had even greater width:height ratios as compared to the outer cones (Two-Way ANOVA- *F*_1, 160_ =  20.436, *P*<0.001). Larger animals and those in better condition were not more ellipsoid-shaped as compared to smaller seals (Multiple Regression- M_T_: *F*_8, 10_ =  18.022, *P* = 0.054, TBL_HTO_: *F*_8, 9_ =  5.268, *P* = 0.325).

**Figure 4:**
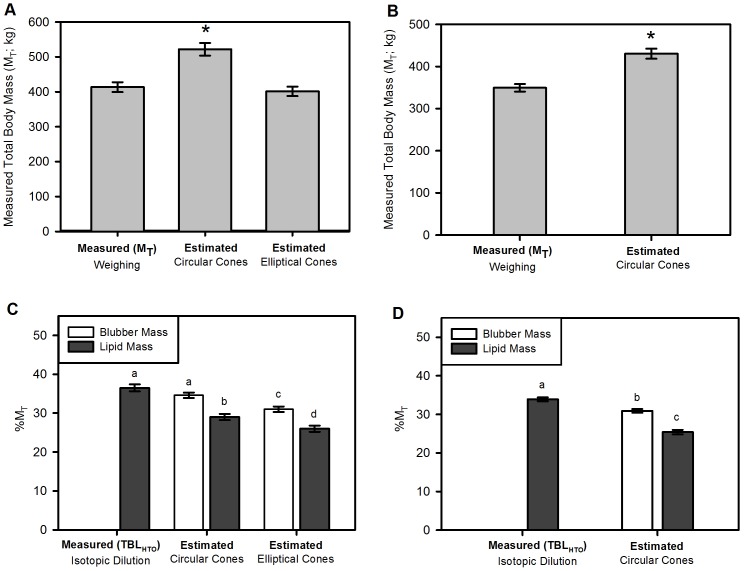
Estimated mass and body composition using truncated cones methods relative to measured values. Mean ± SE estimated total body mass (M_T_) from the “subset study” (**A**; *N* = 10) using both circular and elliptical truncated cones, and (**B**) from the “full study” (*N* = 76) using circular truncated cones. Body composition estimated (**C**) from circular and elliptical cones in the subset study and (**D**) circular cones from the full study are also shown. Blubber with or without corrections for lipid content were compared to total body lipid determined via isotopic dilution (TBL_HTO_). * =  significant difference between estimated and measured M_T_. Different letters =  significant difference between body composition estimates relative to measured lipid stores.

Using standard published values for blubber and body core density, estimated M_T_ using truncated cone calculations with elliptical cross-sections were not significantly different from measured M_T_ (Fig. 4A; Subset Study: Repeated measures ANOVA- *F*_2,18_ =  167.442; Elliptical mean error from M_T_: −11.9±6.8 kg; −2.8±1.7% (range: −8.9 to +7.1%), Bonferroni post hoc- *P* = 0.340). Conversely, estimates using traditional circular cones were significantly higher than M_T_ directly determined by weighing animals (Fig. 4A; Subset Study: Circular mean error from M_T_: 108.4±6.8 kg; 26.3±1.4% (range: +20.6 to +35.3%), Bonferroni post hoc- *P*<0.001; Fig. 4B; Full Study: Circular error from M_T_: 81.0±3.5 kg; 22.8±0.6% (range: +11.3 to +42.2%), Paired t-test, *t*_75_ =  −23.339, *P*<0.001).

As the use of ellipses yielded smaller M_T_ estimates, it also resulted in smaller blubber and core mass estimates. Core body mass estimated using elliptical truncated cones was significantly smaller than estimates using the traditional circular cones (66.2±1.5 vs. 91.7±1.2%M_T_; Paired t-test, *t*_9_ =  19.981, *P*<0.001). The difference in core body mass between the two models was substantially greater than the difference in BM.

### Morphometric versus Isotopic Dilution Estimates of Body Composition

**Figure 5:**
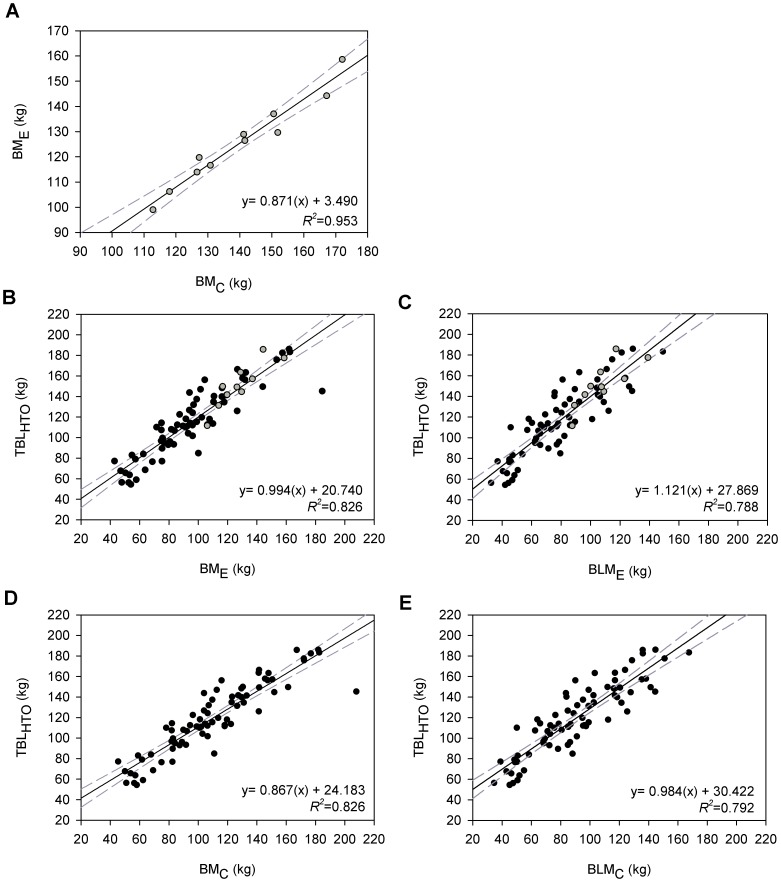
Relationships between morphometric and isotopic dilution body composition results. Linear regression between blubber mass determined using (**A**) elliptical and circular truncated cones (*N* = 11). Once this relationship (*grey*) was used to correct values to elliptical models for additional animals (*black*), regressions were made between lipid mass determined by HTO measurements and elliptical cones with (**B**) and without (**C**) corrections for blubber lipid content. Similar relationships exist when using traditional, circular truncated cones with (**D**) and without (**E**) corrections for blubber lipid content (*N* = 76).

Blubber lipid content determined via biopsy sample (Table 1; range: 61.1−97.4%) was used to convert BM to subcutaneous lipid mass (BLM) for each seal separately. Blubber lipid content at the dorsal umbilicus site was similar to the average blubber lipid content across the body (−2.37% error) in the necropsied seal. All BM_E_, BLM_C_, and BLM_E_ models yielded significantly smaller blubber/lipid masses as compared to isotopic dilution (Fig. 4C; Subset Study: Repeated measures ANOVA- *F*_1.8,16_ =  112.845, Bonferroni post-hoc- all *P*<0.001). The difference between TBL_HTO_ and BLM_E_ was 10.5±0.8% body mass. BM_C_ did not differ from TBL_HTO_ in the study subset, but BM_C_ and BLM_C_ were both significantly lower than TBL_HTO_ in the full study (Fig. 4D; Full Study: Repeated measures ANOVA- *F*_1.5,110.6_ =  190.941, Bonferroni post-hoc- all *P*<0.001). BM_C_ was significantly positively correlated with BM_E_ (Fig. 5A; Subset Study: Regression- *F*_1,9_ =  182.2, *P*<0.001), and this relationship was used to predict BM_E_ for animals in which all measurements could not be taken.

Truncated cones calculations underestimated TBL_HTO_; however, regression models allowed BM to estimate TBL_HTO_. All regressions between morphometric cone models and TBL_HTO_ were highly significant and produced low error (RMSE). Regression errors were similar between elliptical and circular truncated cone models (Fig. 5B-D; Regression- BM_E_: *F*_1,74_ =  351.8, *P*<0.001; RMSE =  14.76 kg, 12.38% TBL_HTO_; BLM_E_: *F*_1,74_ =  274.4, *P*<0.001, RMSE =  16.16 kg, 13.54% TBL_HTO_; BM_C_: *F*_1,74_ =  350.5, *P*<0.001, RMSE =  14.80 kg, 12.41% TBL_HTO_; BLM_C_: *F*_1,74_ =  281.1, *P*<0.001, RMSE =  16.03 kg, 13.44% TBL_HTO_). However, the slope correcting BM_C_ to TBL_HTO_ was significantly < 1 (95% CI: 0.774−0.959) indicating that traditional circular truncated cones underestimated TBL_HTO_ to a greater extent in larger animals. All slopes relating BM_E_, BLM_C_, or BLM_E_ to TBL_HTO_ were not significantly different from 1. Adding season as a variable in regression models allowed for more accurate estimates of TBL_HTO_ from BM (BM_E_: *t_season_* =  2.422, *P* = 0.018, *R* ^2^ = 0.839, RMSE =  14.25 kg, 11.94% TBL_HTO_; BM_C_: *t_season_* =  2.444, *P* = 0.017, *R* ^2^ = 0.839, RMSE =  14.28 kg, 11.97% TBL_HTO_), but adding season did not improve fit between TBL_HTO_ and BLM (e.g. BLM_E_ or BLM_C_).

### Estimating Mass and Body Composition from Regression Models

In the absence of direct M_T_ measurements, the best single morphometric measurements to take in order to estimate M_T_ were sternum (*F*_1,109_ =  794.458, *P*<0.001, *R* ^2^ = 0.879) and middle girths (*F*_1,109_ =  790.550, *P*<0.001, *R* ^2^ = 0.879). Using either of these two girth measurements accounted for 8.8% more variance than using the axillary girth measurement alone to estimate M_T_ (*F*_1,109_ =  412.882, *P*<0.001, *R* ^2^ = 0.791). Adding length measurements to the multiple regression improved model fit and decreased the RMSE. The best model included sternum girth, cLength, middle girth, and sLength, and this estimated M_T_ with RMSE of 16.16 kg or 4.91% M_T_ (Table 4).

**Table 4:** Morphometric measurements to estimate body mass and composition.

**Estimated Parameter**	**Equation**	**AICc**	**ΔAICc**	***R*** **^2^**	**RMSE; kg (%M_T_)**
**Mass (kg)**	4.676×10^−5^ (sLength × Girth:axillary^2^ ) − 11.399	1043.26	11.98	0.896	26.57 (8.08)
Traditional LG^2^ only	4.553×10^−5^ (sLength × Girth:axillary^2^ ) + 18.442(Season) − 10.642	1031.28	0	0.908	25.14 (7.65)
	**Equation**	**AICc**	**ΔAICc**	***R*** **^2^**	**RMSE; kg (%M_T_)**
**Mass (kg)**	4.398(Girth:sternum) − 468.287	1059.47	125.10	0.879	28.34 (8.62)
All morphs	3.003(Girth:sternum) + 1.589(cLength) − 613.603	988.98	54.65	0.937	20.62 (6.27)
	1.443(Girth:sternum) + 1.420(cLength) + 1.565(Girth:middle) − 565.268 *	939.14	4.81	0.961	16.55 (5.03)
	1.509(Girth:sternum) + 0.985(cLength) + 1.497(Girth:middle) + 0.534(sLength) − 580.934 *	934.33	0	0.963	16.16 (4.91)
	**Equation**	**AICc**	**ΔAICc**	***R*** **^2^**	**RMSE; kg (%TBL_HTO_)**
**TBL_HTO_ (kg)**	0.388(M_T_) − 16.258	638.65	44.71	0.793	15.81 (13.25)
M_T_ included	0.349(M_T_) + 20.798(Season) − 12.840	600.6	6.66	0.878	12.29 (10.30)
	0.300(M_T_) + 16.327(Season) + 6.485(Blubb:middle dorsal) − 21.621	593.94	0	0.892	11.87 (9.95)
	**Equation**	**AICc**	**ΔAICc**	***R*** **^2^**	**RMSE; kg (%TBL_HTO_)**
**TBL_HTO_ (kg)**	1.799(Girth:sternum) − 213.980	646.33	38.28	0.771	16.70 (14.00)
M_T_ not included	1.609(Girth:sternum) + 18.227(Season) − 187.788	624.13	16.08	0.834	14.32 (12.00)
	1.305(Girth:sternum) + 8.406(Season) + 8.770(Blubb:sternum lateral) − 165.120	615.25	7.20	0.857	13.82 (11.58)
	0.955(Girth:sternum) + 11.712(Season) + 8.334(Blubb:sternum lateral) + 0.373(cLength) − 195.099	608.05	0	0.874	13.00 (10.90)
	**Equation**	**AICc**	**ΔAICc**	***R*** **^2^**	**RMSE; %M_T_ (%TBL_HTO_)**
**TBL_HTO_ (%M_T_)**	5.152(Season) + 1.287(Blubb:middle dorsal) + 25.749	408.53	0	0.508	3.54 (10.43)

Stepwise forward multiple regressions using morphometric measurements to estimate total body mass (M_T_) and lipid mass (TBL_HTO_; absolute kg and as %M_T_). Factors that were included in each model are shown under the estimated parameter. Each step is shown to elucidate which measurements should be taken preferentially, if animal handling time is limited (all *P*<0.001). * =  Note that the additional parameter in this model had slightly increased the variance inflation factor, and the variance in the coefficients. All lengths, girths, and blubber depths were measured in cm, and when season is a significant parameter, the coefficient should be multiplied by “0” for January and “1” for October study animals. Root-square-mean-error (RMSE) of models is presented as absolute (kg) and as a percentage of the study’s mean M_T_ or TBL_HTO_.

The best model for estimating absolute lipid mass (TBL_HTO_) included M_T_, season, and blubber depth at the middle dorsal site (Table 4; RMSE: 11.87 kg; 9.95% TBL_HTO_). If M_T_ could not be determined in the field, the best model to estimate TBL_HTO_ included sternum girth, season, sternum lateral blubber depth, and cLength measurements (Table 4; RMSE: 13.00 kg; 10.90% TBL_HTO_). The best predictor of TBL_HTO_ (as %M_T_) was season and blubber depth measured at the middle dorsal site (Table 4, RMSE: 3.54%M_T_; 10.43% for %TBL_HTO_).

## Discussion

This study demonstrated the efficacy of the modified elliptical truncated cone model to estimate M_T_ and TBL_HTO_, and showed that a reduced set of non-invasive measurements can be used to estimate these parameters with high accuracy. The traditional truncated cones model using circular animal cross-sections significantly overestimated M_T_ and BM (absolute and as %M_T_), relative to elliptical cones. Still, both the circular and elliptical truncated cone models underestimated lipid stores measured by using isotopic dilution techniques.

That body lipid stores determined by isotopic dilution techniques (TBL_HTO_) are consistently higher than blubber lipid stores, both using elliptical and circular body cross-sections (BLM_E_ and BLM_C_), suggests Weddell seals have significant internal lipid deposits that would be overlooked by solely using morphometric measures of condition. Previous work has demonstrated the presence of intramuscular lipid reserves and lipid sheaths around internal organs and abdominal mesentery [18], [20], . These internal stores may be mobilized first during times of reduced foraging [52], and would also impact the animal’s net buoyancy and cost of locomotion [14], [53]. Therefore, ignoring internal lipid reserves could introduce biases when comparing body composition among species, populations, and seasons.

The errors in Weddell seal M_T_ estimates using traditional circular truncated cones were not substantially improved when blubber and core tissue densities were slightly altered. Only if the total body average density of Weddell seals was assumed to be 0.83±0.01 g·mL^−1^ were estimates of M_T_ equal to measured values. This density is well outside of the physiologically-relevant range, as measured blubber densities are 0.92−0.95 g·mL^−1^ [29], [54], and the average densities of lean mass components for mammals are approximately 1.1 g·mL^−1^ [29], [30], [44], [55]. In contrast, elliptical truncated cones provided estimates of M_T_ that were not significantly different from measured values when using published blubber and core density estimates to convert body volume to mass. Further, elliptical models did not require additional empirically-determined correction factors to accurately estimate M_T_.

The fact that elliptical, but not circular, truncated cones closely approximated actual M_T_ indicates that a major source of error in the traditional truncated cones method is the assumption that animals are circular in cross-section. Elliptical cross-sections much more accurately reflect the animals’ true body shape while hauled-out and lying flat against the ice. This deformation, or “slumping,” was first described by Slip et al. [34], and was supported by the fact that field measurements of sculp mass were smaller than estimates using the traditional circular truncated cones calculations [56]. However, this study is the first, to our knowledge, to demonstrate that the compression of the core body mass into non-circular form introduces error. Using circular truncated cones to estimate core body mass produced values that were much too high, at 91.7% M_T_. These errors arise because circles have the largest area per unit arc length, and therefore, the greater the degree of asymmetry of the animal (i.e. greater width:height ratio), the larger the overestimate in cross-sectional area.

The errors accompanying M_T_ and BM estimates from circular cones are likely to be important when calculating animal drag forces, buoyancy, density, and metabolic costs; all of which are influenced by surface area and volume calculations (Table 5). Conversely, variations between circular and elliptical models likely would not impact the relative differences and trends when simply comparing body condition within a population. Thus, either method could be used as an ecosystem metric or index, provided it is understood that circular truncated cones are not yielding accurate estimates of mass or body composition unless additional correction factors are included in the model.

**Table 5:** Differences in additional physiological parameters determined by circular vs. elliptical truncated cones.

Physiological Estimate	Circular Cones	Elliptical Cones
Total Volume (L)*	490.3±17.3	379.8±13.4
**Surface Area (SA; cm^2^)**	3773.4±93.2	3773.4±93.2
SA:V*	7.73±0.10	9.98±0.15
Net Buoyancy (N)*	−57.3±5.0	−76.2±5.7
**Calculated Density (g·mL** ^−**1**^ )*	0.83±0.01	1.07±0.02

Mean ± SE total body volume, surface area, surface area-to-volume ratios, buoyant force, and calculated density in Weddell seals (*N* = 11). Surface area remains the same between circular and elliptical cones. Buoyancy was calculated following Webb et al. [9], and density was calculated using measured M_T_. *Asterisk* indicates a significant difference in estimated parameter using circular versus elliptical models (paired t-tests; all *P*<0.001).

This study indicates that animal M_T_ and BM can be accurately estimated with volumetric and morphometric models, but that accurate estimates of lipid stores require isotopic dilution techniques or additional calibration factors. In combination, direct weighing and isotopic dilution techniques were found here to be the most appropriate tools when precise measures of animal size, body composition, or energetic costs are required. As this is not always possible in a field setting, there is a need for predictive models that relate proxy variables, such as morphometric measurements, to mass or body composition [34], [40]. Such models do not make assumptions about animal shape, but instead rely on a “calibration dataset” for coefficient development. This study has shown that, once developed, these simplified models can be used to estimate mass and body composition quickly for a much larger sample size. Moreover, they can be quite accurate (RMSE: 4.91% for M_T_; 10.90% for TBL_HTO_; 10.43% for %TBL_HTO_), and require much less time and effort as compared to direct measures or the more complex truncated cones models. Indeed, the multiple linear regression approach predicted body composition more accurately than the truncated cone models, while using far fewer morphometric measurements. Surprisingly, the models that produced the most accurate estimate of M_T_ included sternum rather than the axillary girth measure, which has traditionally been incorporated into the straight length × axillary girth^2^ (LG^2^) proxy [26], [27]. Similarly, the model that produced the most accurate absolute and percent TBL_HTO_ used middle dorsal or sternum lateral blubber depth measures, respectively, instead of the traditional single axillary blubber measurement [28].

Identifying suitable proxy variables using hierarchical regressions can lead to reduced handling times and simpler procedures; however, predictive power will depend on model development using test datasets. This is because the relationship between morphometric measures with animal M_T_ and TBL_HTO_ are likely to be species-specific and vary seasonally. In contrast, the elliptical truncated cones method does not require such calibration coefficients and is; therefore, more broadly applicable and useful in new species and field situations. In addition to utilizing morphometric measurements to estimate M_T_, there have been some recent successes in photogrammetric methods. While these techniques to estimate M_T_ have been validated within <2−10% accuracy in pinnipeds and allow researchers to avoid animal handling [57]–[59], photogrammetry can’t quantify total body lipid stores. Alternatively, dive loggers have been used to estimate net animal buoyancy by measuring changes in animal drift rates through the water column. Since buoyancy is influenced by total body lipid content (both in the blubber and internal stores), drift rates are used as a proxy of changes in body composition [53], [60], [61]; however, this method doesn’t provide estimates of M_T_. The optimal technique for determining animal mass and body composition clearly depends on multiple factors such as handling and analytical constraints, whether precise or index values are needed to accomplish study goals, and the availability of test datasets for development of appropriate correction factors.
